# Effect and mechanisms of reproductive tract infection on oxidative stress parameters, sperm DNA fragmentation, and semen quality in infertile males

**DOI:** 10.1186/s12958-021-00781-6

**Published:** 2021-06-28

**Authors:** Kang-Sheng Liu, Xiao-Dong Mao, Feng Pan, Rui Fang An

**Affiliations:** 1 Department of Obstetrics and Gynecology, The First Affiliated Hospital of Xi’an Jiaotong University, Xi’an, Shannxi 710061 China; 2 Department of Clinical Laboratory, Women’s Hospital of Nanjing Medical University, Nanjing Maternity and Child Health Care Hospital, Nanjing, 210029 Jiangsu China; 3 Department of Endocrinology, Affiliated Hospital of Integrated Traditional Chinese and Western Medicine, Nanjing University of Chinese Medicine, Nanjing, 210028 Jiangsu China; 4 Department of Andrology, State Key Laboratory of Reproductive Medicine, Women’s Hospital of Nanjing Medical University, Nanjing Maternity and Child Health Care Hospital, Nanjing, 210029 Jiangsu China

**Keywords:** DNA fragmentation test, Ureaplasma urealyticum, Chlamydia trachomatis, Semen parameters, Malondialdehyde, Total antioxidant capacity; single-cell gel electrophoresis; SCD test

## Abstract

Recent years have seen a rising incidence of male infertility, mostly caused by the decline of sperm quality. The ratio of infertile males to infertile females has escalated from 3:7 in 2013 to current 5:5, which turns male infertility into the research focus of reproductive medicine. This study aimed to clarify the effect of reproductive tract infection by ureaplasma urealyticum (UU) and chlamydia trachomatis (CT) on the DNA integrity and routine semen parameters of infertile males. A retrospective study was performed. A total of 259 infertile males who were treated at the Andrological Laboratory Examination and Reproductive Medicine Center in our hospital were analyzed. qRT-PCR was used to examine the infection status of CT and UU. According to the eligibility criteria, we evaluated the semen parameters and biochemical data of 253 men. Based on the results of PCR, the subjects were divided into four groups: Group I (CT positive, 63 cases), Group II (UU positive, 60 cases), Group III (CT positive and UU positive, 62 cases), and Group IV (no infection, 68 cases). DNA fragmentation index (DFI), sperm count, vitality and morphology, elastase level, seminal plasma malondialdehyde (MDA), and total antioxidant capacity (TAC) were assessed. Compared to Group IV, three groups (Group I, Group II and Group III) showed difference in semen volume, proportion of sperm with normal morphology, sperm motility, progressive motility, and vitality (*P <* 0.05). Compared to Group IV, Group II and Group III showed difference in DFI (*P <* 0.05). Compared to Group IV, Group II and Group III showed difference in elastase level *(P <* 0.05). VCL, VSL, VAP, WOB, ROS, TM, HDS showed differences between groups of abnormal/normal WBC (*P < 0.01).

UU infection significantly increased the level of seminal leukocytes only in Group II, but not in the other three groups, indicating that UU is a factor to increase the level of seminal leukocytes. Compared with the normal leukocyte group, there were significant differences in total motility, forward motility and normal sperm ratio between the two groups. The proportion of sperm with abnormal morphology (mostly in the head) showed obvious difference between groups of high and normal seminal leukocytic levels. At the same time, in this study, SCGE and SCD verified that leukocytes could damage sperm DNA by increasing ROS, which ultimately affects male fertility.

## Introduction

Human sperm quality can be affected by various factors, including physical injury, psychological stress, or environmental pollutants [1]. Reproductive tract infection, usually presented by non-gonococcal urethritis, is often caused by ureaplasma urealyticum (UU) and chlamydia trachomatis (CT) [2]. Male reproductive tract infection, a big threat to reproductive health, is responsible for the infertility in about 15% patients [3]. Gonococcal urethritis caused by these two types of organism is more likely to invade male’s genital tract, epididymides, prostate gland, and testes, exerting bad effect on semen quality, spermatogenesis, sperm-egg binding, and pregnancy outcomes [4]. Mycoplasma infection and other causes of chronic prostatitis may also damage sperm DNA [5]. UU, a pathogenic microorganism, can adhere to the enitourinary tract and evoke urinary tract infections. It can also affect male fertility in several ways, such as interfering with spermatogenesis, distorting sperm metabolism and sperm-egg binding, as well as triggering immune responses in the genital tract [6]. If male reproductive tract infection develops, a large amount of elastase escapes out of the cell and interact with other oxidants to fulfill its anti-inflammatory role. Elastase, a kind of soluble protease, is evenly distributed in the semen . It has been reported that reproductive tract infection can increase the ROS level in semen, cause lipid peroxidation of sperm membrane and sperm nuclear damage, and the mechanism may involve the increase of white blood cells in semen [7] MDA is one of the degradation products of lipid peroxidation. Detection of the change of its relative content can help us understand the peroxidation of biofilm, and indirectly reflect the damage degree of ROS to the structure and function of reproductive system and sperm membrane. TAC represents the overall level of antioxidant capacity in the body, and the antioxidant capacity can be reflected by measuring the content of TAC in the seminal plasma [8, 9].

The methods to detect the two organisms are in continuous renewal. Samples detected with the RNA analytical method far outnumber those with the DNA analytical method, for the former is more specific, precise and time-saving [10]. PCR analysis has a high specificity up to 99–100%, but its false negative results may result from the “carry over” and false positive results from Taq inhibitors in the samples [11]. Multiple PCR shows a sensitivity and specificity close to 100% in UU detection [12]. Nucleic acid hybridization test has a sensitivity of 70–92% and a specificity of 97–98% in detecting CT specific RNA [13]. CT DNA can also be detected by PCR [14]. Nucleic acid amplification test can detect the sequences of CT DNA or RNA, with a sensitivity > 90% and a specificity > 99%. This test has also been approved by the FDA to screen and diagnose CT infection [15]. Besides, sperm DNA fragmentation analysis has been developed to evaluate sperm quality and fertility. This method is more accurate than the routine semen analysis, the efficiency of which is affected by multiple factors [16]. Human sperm DNA is the carrier of hereditary information of humans. Sperm DNA damage is partly associated with male infertility [17]. The normal reproductive processes, including sperm-egg fusion, membrane fusion, chromosome combination, and embryogenesis, can only be accomplished by sperm with intact DNA [18]. Up to now, few researchers have used DFI to examine the effect of two organisms on male infertility in China. Therefore, this study was designed to explore:

1. The impacts of different groups of reproductive tract infections (I,II,III,IV) on sperm parameters and leukocyte counts in semen.
2. The impacts of leukocytes on sperm parameters, motion parameters and sperm morphology.
3. The effects of reproductive tract infection on oxidative stress parameters, sperm DNA fragmentation, and semen quality in infertile males.

## Materials and methods

### Study population

According to the definition of male infertility set by the WHO (Definition of infertility: Failing to fertilize the wife after more than 12 months’ cohabitation and normal sex without any contraceptive intervention (5th ed. Beijing, People’s Health Press) [19], a total of 259 infertile males (ages18 to 35 years, mean age 31 + 2.3 years) who were treated at the Andrological Laboratory Examination and Reproductive Medicine Center at our hospital in 2016 and 2017 were included. Excluded were those with trauma, family diseases, urological and reproductive diseases (like varicocele, cryptorchidism, prostatitis, epididymitis) sexual dysfunction, medication history (hormones or cytotoxic drugs) and occupational exposure (to zinc, high-density radiation, chemicals, high temperature).

Varicocele was diagnosed with ultrasound covering the vessels of pampiniform plexus; no conceiving was observed after > 2 years of regular sexual intercourse (causes from the woman were excluded); ultrasound showed dilated unilateral or bilateral spermatic vein of > 2 mm in diameter; valsalva maneuver reduced venous return; palpation showed obvious thickening in spermatic vein with/without ipsilateral testicular atrophy; normal semen parameters or sperm quality [20].

**Fig. 1:**
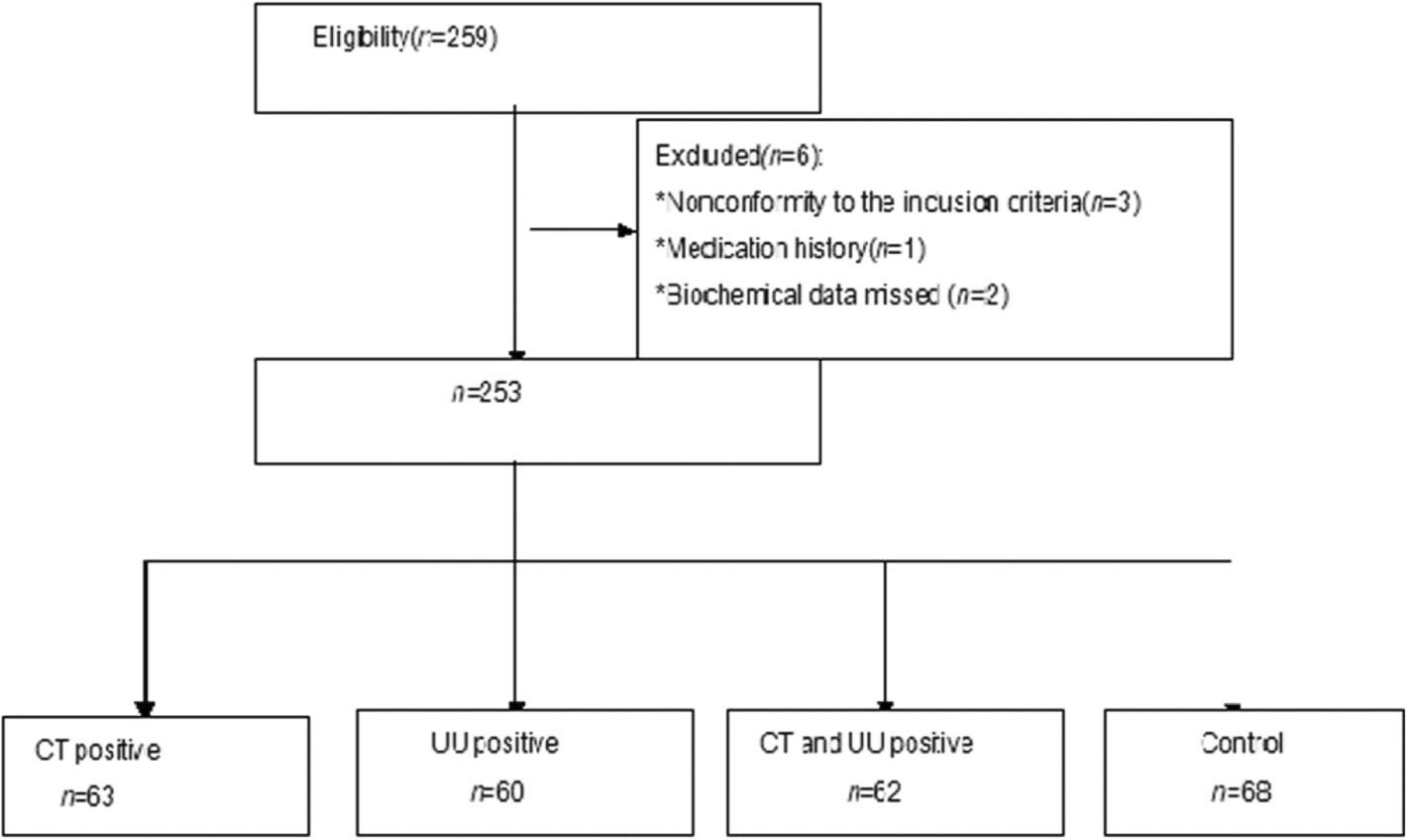
The flowchart of baseline characteristics of participants. After fulfilling the eligibility criteria, we evaluated the semen parameters and biochemical data of 253 men. According to results of PCR, all the subjects were divided into four groups: group I (CT positive, 63 cases), Group II (UU positive, 60 cases), Group III (CT positive and UU positive, 62 cases), and Group IV (no infection, 68 cases).Defnitions:CT = chlamydia trachomatis; UU = ureaplasma urealyticum

Three semen samples were excluded for their nonconformity to the inclusion criteria (one for medication history and two for missed biochemical data). Next, we evaluated the semen parameters and biochemical data of 253 males. The baseline characteristics are presented in Fig. 1. According to the results of CT-DNA and UU-DNA tests, all the patients were divided into four groups: Group I (CT positive, 63 cases), Group II (UU positive, 60 cases), Group III (CT positive and UU positive, 62 cases), and control Group IV (no infection, 68 cases). Patients in the four groups showed no difference in age and the duration of infertility.

**Fig. 2:**
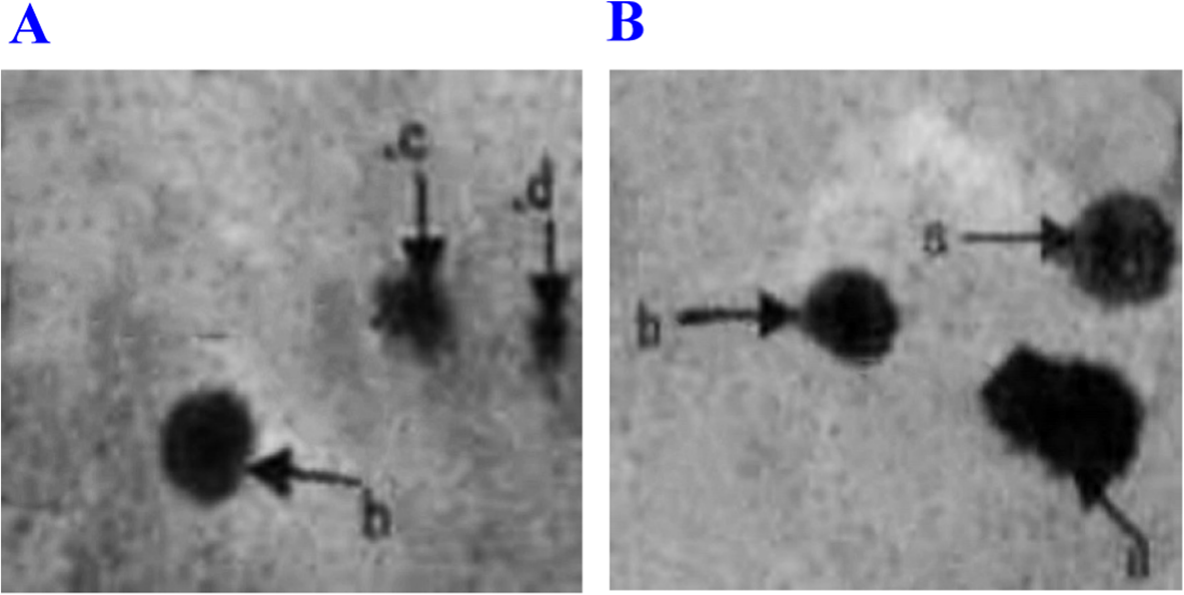
Ordinary optics microscope images of sperm in SCD test (Switzer land staining 10 × 20). **A** Sterile patients with mycoplasma infection; **B** Males with normal fertility.a. large-size dispersion halos; b. medium-size dispersion halos; c. small-size dispersion halos; d. no dispersion halo. The percentages of sperm small-size dispersion halos and no dispersion halo were significantly higher in Group A than those in Group B. On the contrary, the percentage of sperm with large-size dispersion halos was significantly lower in Group A than that in Group B (*P* < 0.05)

### Ethics

The research was approved by the Ethics Committee of Women’s Hospital of Nanjing Medical University, and was conducted in accordance with the Declaration of Helsinki. An information sheet was provided to all participants. Written informed consent was obtained from all participants. The relevant guidelines and regulations of the local institute were strictly followed. Participants could withdraw from the trial without giving a reason.

#### Sample collection

##### Semen collection

Semen samples were collected by masturbation after 2 to 5 days of ejaculatory abstinence (WHO, 2010) [19]. The duration of abstinence was recorded. Each semen sample was directed into a sterile plastic cup, liquefied in an incubator at 37 °C. After the semen was completely liquefied, at least 2.6 ml of semen was taken for the test (1.5 ml of the semen was used for routine semen analysis, the left for DNA fragmentation analysis and oxidative stress parameters). Semen in the sterile tube was also used for CT-DNA and UU-DNA tests.

##### Routine semen analysis

According to the Laboratory Manual of the WHO for the Examination and Processing of Human Semen (5^th^ edition) and the WHO Manual for the Standardized Investigation, Diagnosis and Management of the Infertile Male [21], the routine semen analysis was performed with a semen quality detection system (WLJY-9000, Beijing Weili New century Science & Tech Dev. Co. Ltd) and supporting reagents. Main parameters were as follows [22]. Image acquisition frame: low and middle sperm concentration collected at 20 Hz, and high sperm concentration at 7 Hz; acquisition interval: 3 ms; maximum sperm motile velocity: 200 um s^-1^; area range of spermatozoa head detected at 7-60 um^2^. Index of sperm motility: straight line velocity (VSL). Grayscale thresholds were set to collect spermatozoa and exclude nonsperm granules. According to the thresholds set for sperm analysis, sperm images were collected and analyzed.

##### Sperm morphology assessment

For morphological evaluations, seminal smears were stained with Diff-Quik (MICROPTIC S.L. Co., Barcelona, Spain) [23]. Approximately 10 μl of sperm was smeared into a thin and homogeneous layer on a clean glass slide and was air-dried at room temperature for at least 10 min. The slides were stained and observed under a brightfield microscope (BH-2; Olympus, Tokyo, Japan) at 1000 × magnification. According to WHO guidelines, a sperm with deformed head, or midpiece, or principal piece was counted as. SDI (sperm deformity index) = number of deformed sperm/number of total sperm^22^. For each semen sample, at least 200 sperm (or the whole sperm if the slide had less than 200 sperm) were counted via a double-blinded method. Then, the percentage of sperm with normal morphology was calculated.

##### DFI (SCD test)

Using the SpermFunc™ DNAf kit (BRED Life Science, Shenzhen, China), SCD test was performed to measure the DNA fragmentation in native and DGC-separated semen. Gelled aliquots of low-melting-point agarose in the kit were provided for semen sample processing in Eppendorf tubes. Eppendorf tubes were placed in a water bath at 80 °C for 20 min to melt the agarose, then transferred to a water bath at 37 °C for 5 min for temperature equilibration. A total of 60 μL of sampled semen was added to and mixed with the agarose in the Eppendorf tubes. Then, 30 μL of semen-agarose mixture was pipetted onto precoated slides in the kit that were covered with a 22 × 22-mm coverslip. The slides were placed on a cold plate in the refrigerator (4 °C) for 5 min, allowing the agarose to produce microgel in which the sperm cells were embedded. The coverslips were gently removed, and the slides were immediately immersed horizontally in solution A and incubated for 7 min. Next, the slides were horizontally immersed in solution B for 25 min. After being washed for 5 min in a tray with abundant distilled water, the slides were dehydrated in gradient concentrations of ethanol (70, 90, 100%; respectively) for 2 min, air-dried, and stored at room temperature in opaque closed boxes [24].

For bright-field microscopy, the slides were horizontally covered with a mixture of Wright’s staining solution (BRED Life Science, Shenzhen, China) and phosphate buffer solution (BRED Life Science, Shenzhen, China) (1:2) for 15 min with continuous airflow. Then, the slides were washed in running water for 10 s and allowed to dry. Strong staining was recommended to allow the periphery of the dispersed DNA loop halos more visible. A minimum of 500 sperm were counted on each sample under the 100X magnification [25].

Normal spermatic DNA presented radiate halos and damaged spermatic DNA presented no or small halos. Fragmented sperm referred to those having a small or no halo. The thickness of the halo on one side was less than the 1/3 diameter of the head’s thinnest part [15]. The rate of sperm DNA fragmentation (%) = the number of sperm with fragmented DNA ÷the total number of sperm × 100%, and < 25% was considered normal [24].

##### Determination of sperm DNA damage by SCGE (single-cell gel electrophoresis)

The semen samples were centrifuged at 800 g at room temperature for 5 min, and resuscitated in PBS. The cell concentration was adjusted to 2 ×  10^6^ /mL. Then, 85 μL of 1% agar-agar with normal melting point (excluding Ca^2+^, Mg^2+^, 50 °C) was laid on a completely frosted glass slide, covered with cover glass, and placed in a refrigerator at 4 °C for 10 min until being solidified. After removal of the cover glass, 10 μL sperm suspension was mixed with 75 μL agarose (0.5%, low melting point) at 37 °C, spread on the slide as the second layer, covered with the glass and refrigerated at 4 °C for 10 min until being solidified. After removing the cover glass, 75 uL of agarose (0.5%, low melting point, 37 °C) was spread as the third layer, and refrigerated at 4 °C for 10 min. Then, cover glass was removed again and the slides were immersed in 4 °C precooled lysate (2.5 mol/L NaCl, 100 mmol/L Na_2_EDTA, 10 mmol/L Tris buffer, 1% sodium dodecyl sarcosine, pH = 10, and the final concentration before use was 1% Triton x-100 and 10% DMSO) for 1 h. The slides were removed from the lysis solution and treated with 10 mmol/L dithiosetanol (DTT) at 4 °C for 30 min, followed by 4 mmol/L of lithium diiodine salicylate (LIS) at 20 °C for 90 min. After rinsing with the electrophoretic solution, the slides were placed in the horizontal electrophoresis tank. The electrophoresis tank was filled with freshly prepared pre-cooled electrophoretic solution (1 mmol/L Na_2_EDTA, 300 mmol/L NaOH), which was over the glass slides (about 0.25 cm above the slides), and placed for 20 min to make the DNA unscrew. Electrophoresis was performed at room temperature for 20 min (1 V/cm, 300 mA). After electrophoresis, 0.4 mol/L Tris (pH 7.5) was used for 3 times, 10 min each time. After staining with 40uL ethidine bromide (EB, 5 μg/mL), the slides were immediately observed and photographed under an inverted fluorescence microscope at 20X magnification (laser wavelength 515–560 nm, blocking wavelength > 590 nm). Fifty cells were randomly selected from each slide to measure the tail moment (TM, the percentage of tail DNA in the total DNA multiplied by tail length) [7].

##### Seminal malondialdehyde and total antioxidant capacity measurement

Spectrophotometry was used to test the level of TAC (U/L) and MDA (nmol/mL). MDA levels were determined using the thiobarbituric acid (TBA) method. Semen samples were centrifuged at 4 °C for 15 min with a speed of 2000 r/min. The supernatant was mixed with the reagents supplied in an MDA Assay Kit (Nanjing Jiancheng Bioengineering Corporation, China, A003–2) and incubated at 95 °C for 40 min. Having been cooled at room temperature, the mixture was centrifuged at 4000 g for 10 min. The absorbance of the supernatant was measured at 530 nm. All operations were performed according to the manufacturer’s instructions. The MDA concentrations were expressed as nmol/mL [26].

Seminal elastase detection was conducted with Microplate Reader (RT-6000) and kits (Huakang Co. Ltd., Shenzhen, China). First, the seminal plasma was separated from the liquefied semen through centrifugation and diluted with a ratio of 1: 51 (10 of UL vs. 500 UL of solution). The ELISA wells were coated with goat anti-elastase antibodies to trap α-1-antitrypsin complex. After being washed, the wells were added with enzyme-labeled antibodies against α-1-antitrypsin. After being incubated and washed and substrate showed observable color, the absorbance was measured. Results: normal (<290 ng/ml), inapparent infection (290–1000 ng/ml), infection (>1000 ng/ml) [27].

##### Detection of sperm ROS

Luminol was used as the probe to detect the products. After being washed by PBS, the sperm cell concentration was adjusted to 2 × 10^7^/mL. Then, 10 μL of luminol dissolved in DMSO (5 mmol/L) was added to the sperm suspension (400 μL) and the fluorescence values were recorded immediately. Another 5 μL of luminol was added into 400 μL of PBS solution as blank control. A mixed suspension of 25 ml H_2_O_2_ with 10 μL luminol was used as positive control. The fluorescence signal was measured using a fluorescence spectrophotometer for 15 min. The result was presented in relative fluorescence unit [28].

##### CT-DNA and UU-DNA tests

CT-DNA and UU-DNA tests were performed with PCR and kits (Daan Co. Ltd., Guangzhou, China). qRT-PCR was performed to determine NG-DNA, CT-DNA, and UU-DNA levels. Using kits (DAAN GENE, Guangzhou, China) and cycler Prism 7500 (ABI, USA), CT-DNA and UU-DNA tests were carried out: 93 °C for 2 min, 1 cycle; 93 °C for 45 s and 55 °C for 60 s, 10 cycles; 93 °C for 30 s and 55 °C for 45 s, 30 cycles. For each experiment, the standard curve was generated according to the standard in the kit. Negative and blank control groups were set. The quantified data was software-analyzed. The level of pathogen ≥500 copy/ml was considered positive, while < 500 copy/ml was referred as negative [29].

##### Leukocyte concentration

Leukocyte concentration was determined after special peroxidase staining (Shenzhen Huakang, China). For staining, 0.1 mL of semen was added into the centrifuge tube, and the staining solution (1 mL) was mixed into the wells, shaken for 2 min, rested at room temperature for 20–30 min, then shaken and mixed the wells. A wet smear was prepared and observed under a microscope at high magnification. Leukocytes of peroxidase-positive were stained brown, and peroxidase-negative cells were not stained. According to the number of leukocytes, the samples were divided into the semen leukocyte normal group (semen leukocyte < 1 × 10^6^/L) and the semen leukocyte abnormal group (semen leukocyte > 1 × 10^6^/L) [13].

### Statistical analysis

SPSS (19.0) was used for statistics analyzes. Chi-square test was used for comparison between proportions. *P* value corrected with a’ = a/2(k^− 1^) was used to compare the proportions. Comparison between multiple groups was performed using ANOVA, and comparison between two groups with LSD. Data shown as mean ± standard deviation were analyzed with t test. The homogeneity of variances was determined with Leven s test. Variables with low homogeneity were treated with Mann-Whitney test. *P <* 0.05 was considered statistically significant.

## Results

### The impacts of different groups of reproductive tract infections on sperm parameters

We compared the semen parameters in 253 patients. All the parameters from group I, II, and III were significant different from those of the control group (*P <* 0.05). Three parameters (proportion of sperm with progressive motility, vitality and motility) of Group III were significantly lower than those of Group I and II (*P <* 0.05) (Table 1).

**Table 1:** Comparison of semen parameters in the four reproductive tract infections groups

Semen parameters	I (***n*** = 63)	II (***n*** = 60)	III (***n*** = 62)	IV (***n*** = 68)
Proportion of sperm with progressive motility (%)	39.8 ± 10.6 ^a^	39.9 ± 13.2^a^	35.2 ± 11.3 ^a,b^	59.2 ± 6.2
Vitality	61.2 ± 10.3 ^a^	62.3 ± 13.6 ^a^	51.4 ± 11.4 ^a,b^	81.2 ± 6.3
Motility (%)	46.8 ± 9.1 ^a^	46.3 ± 11.2^a^	41.2 ± 10.5 ^a,b^	72.2 ± 5.0
Volume (ml)	2.6 ± 0.9 ^a^	2.8 ± 1.2 ^a^	2.7 ± 1.3^a^	3.8 ± 1.5
Proportion of sperm with normal morphology (%)	3.9 ± 1.8^a^	2.9 ± 1.6 ^a^	3.2 ± 1.7 ^a^	4.2 ± 1.7
Sperm count (million per milliliter)	102.5 ± 87.2 ^a^	93.1 ± 91.3 ^a^	91.6 ± 83.5 ^a^	136.6 ± 81.2

Group I (CT positive), Group II (UU positive), Group III (CT positive and UU positive), Group IV (control). Mann-Whitney was used for comparison of sperm count

^a^Compared with Group IV. *P < 0*.05

^b^Compared with Group I, II. *P* < 0.05

**Fig. 3:**
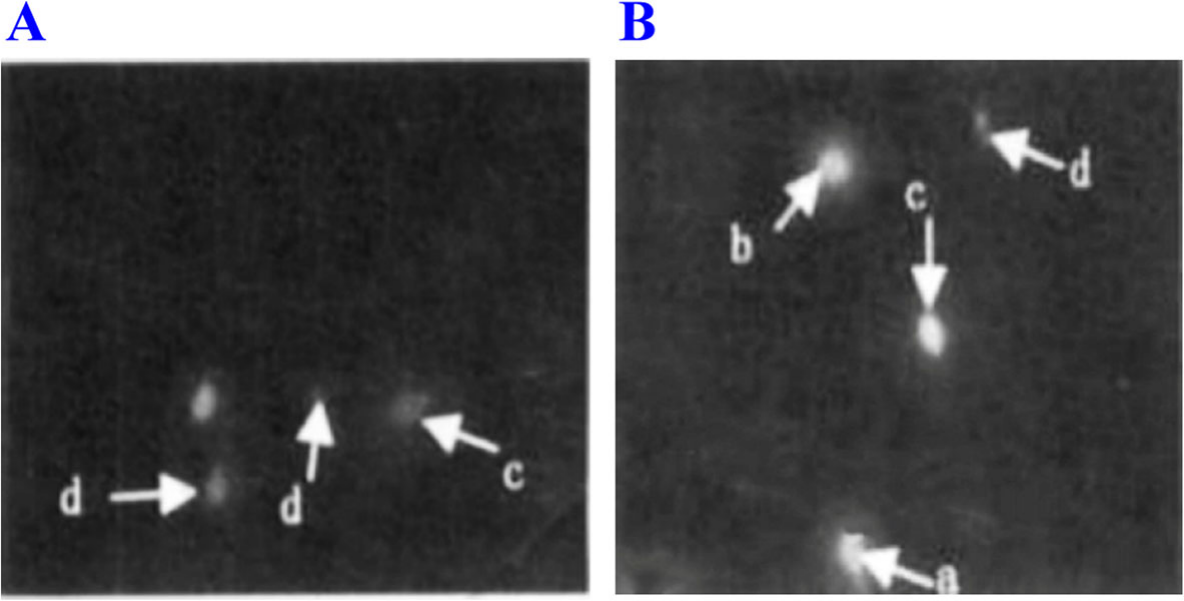
SCD test, AO staining for fluorescence microscopy (AO staining 10 × 40). **A** Sterile patients with mycoplasma infection; **B** Males with normal fertility. a. large-size dispersion halos; b. medium-size dispersion halos; c. small-size dispersion halos; d. no dispersion halo. The percentages of sperm small-size dispersion halos and no dispersion halo were significantly higher in Group A than those in Group B. On the contrary, the percentage of sperm with large-size dispersion halos was significantly lower in Group A than that in Group B (*P <* 0.05)

### The effects of different groups of reproductive tract infections on sperm DNA fragmentation, seminal elastase level and oxidative stress parameters

We detected the infection among 63 patients in Group I (CT Positive), 60 patients in Group II (UU Positive), 62 patients in Group III (CT Positive and UU Positive) and 68 patients in Group IV (Control). Two parameters (DFI,elastase) of Group II and III were significantly higher than those of Group IV, with statistical significance (*P <* 0.05) (Table 2). Group I, Group II and Group III showed parameters (MDA, TAC) different from those of Group IV *(P*<0.05) (Table 2).

**Table 2:** Comparison of sperm DFI, seminal elastase level and oxidative stress parameters in different groups

Parameters	I (***n*** = 63)	II (***n*** = 60)	III (***n*** = 62)	IV (***n*** = 68)
DFI (%)	19.1 ± 9.2	26.5 ± 12.3 ^a^	30.3 ± 15.6 ^a^	15.6 ± 8.9
Elastase (ng /ml)	565.2 ± 366.7	920.6 ± 751.2 ^a^	1241.8 ± 1016.5 ^a^	256.5 ± 172.6
MDA (nmol/ml)	3.6 ± 0.2^b^	5.0 ± 0.2 ^b^	5.1 ± 0.3 ^b^	1.7 ± 0.2
TAC(U/L)	19.1 ± 2.8 ^b^	18.5 ± 1.5 ^b^	16.0 ± 2.2 ^b^	31.3 ± 5.2

*DFI* DNA fragmentation index, *MDA*:malondialdehyde, *TAC* total antioxidant capacity

^a^Group II and Group III compared with Group IV, *P* < 0.05 (Fig. 2, Fig. 3)

^b^Group I, II, III compared with Group IV, *P <* 0.05

**Table 3:** Proportions of patients with increased seminal leukocytic level in four groups

Group	I (***n*** = 63)	II (***n*** = 60)	III (***n*** = 62)	IV (***n*** = 68)	***x***^**2**^	***P***
WBC > 10^6^ (*n* = 53)	10/63 (15.8%)	20/60 (33.3%)*	17/62 (27.3%)	6/68 (8.8%)	12.6	0.005*
WBC < 10^6^ (*n* = 200)	53/63 (84.2%)	40/60 (66.7%)	45/62 (72.7%)	62/68 (91.2%)		

Group I (CT positive), Group II (UU positive), Group III (CT-UU-positive), Group IV (control)

Chi-square test was used for comparison. *P* value corrected with a’ = a/2(k^−1^) was used to compare the proportions. *P <* 0.05 was considered statistically significant (*P < 0.01) (Table 3)

### Proportions of patients with increased seminal leukocytic level in four groups

The effect of infection on white blood cells in semen was studied. The white blood cells increased in 10 of 63 people in Group I, 20 of 60 people in Group II, 17 of 62 people in Group III, and 6 of 68 people in Group IV. There was statistical difference in the proportion of patients with abnormal white blood cells among the four groups (x^2^ = 12.6, *P* = 0.005). The proportion was statistically different between Group II (33.3%) and Group IV(8.8%).

### Seminal parameters changed as seminal leukocytic level increased

Seminal parameters in groups of abnormal/normal leukocytic levels were compared with t-test. Sixty normal semen samples were randomized as the control. The results showed different total motile counts, progressive motility, and proportions of normal-morphology sperm. *P* < 0.05 was considered statistically significant (*P < 0.01) (Table 4).

**Table 4:** Sperm parameters varied as the seminal leukocytic level increased

Parameters	WBC < 10^**6**^ (***n*** = 60)	WBC > 10^**6**^ (***n*** = 53)	t	p
Volume (ml)	2.9 ± 1.1	2.8 ± 0.9	0.2	0.815
PH	7.3 ± 0.2	7.3 ± 0.2	0.8	0.301
Sperm count (× 10^6^/ml)	76.2 ± 41.2	75.1 ± 30.2	1.2	0.213
Total motile sperm count, % (× 10^6^/ml)	53.7 ± 25.2	45.3 ± 26.2	2.6	0.008*
Count of sperm with good progressive motility (PR%)	46.2 ± 15.1	38.3 ± 13.4	2.7	0.006*
Proportion of sperm with normal morphology (%)	4.4 ± 1.5	2.9 ± 1.0	1.1	0.005*
VCL (um/s)	46.2 ± 9.0	41.3 ± 9.1	2.2	0.031*
VSL (um/s)	29.5 ± 6.2	26.2 ± 6.1	2.6	0.015*
VAP (um/s)	30.3 ± 6.8	25.0 ± 6.4	2.3	0.018*
ALH (um)	4.0 ± 2.1	4.1 ± 2.1	0.4	0.656
WOB (%)	68.6 ± 5.2	63.3 ± 5.2	3.6	0.002*
Log (ROS + 1)	1.8 ± 0.8	2.9 ± 0.6	6.3	0.001*
TM	0.9 ± 0.2	1.2 ± 0.6	3.8	0.009*
HDS (%)	6.5 ± 3.1	11.6 ± 5.1	4.1	0.028*
MDA (nmol/ml)	2.6 ± 0.3	4.5 ± 0.5	0.3	0.035*
TAC (U/L)	23.1 ± 3.3	15.1 ± 1.6	3.5	0.020*
DFI (%)	20.0 ± 10.2	28.2 ± 13.2	2.8	0.022*

*DFI* DNA fragmentation index, *VCL* curvilinear velocity, *VSL* straight-line velocity, *VAP* average path velocity, *ALH* amplitude of lateral head displacement, *WOB* wobble, *TM* tail moment

**Fig. 4:**
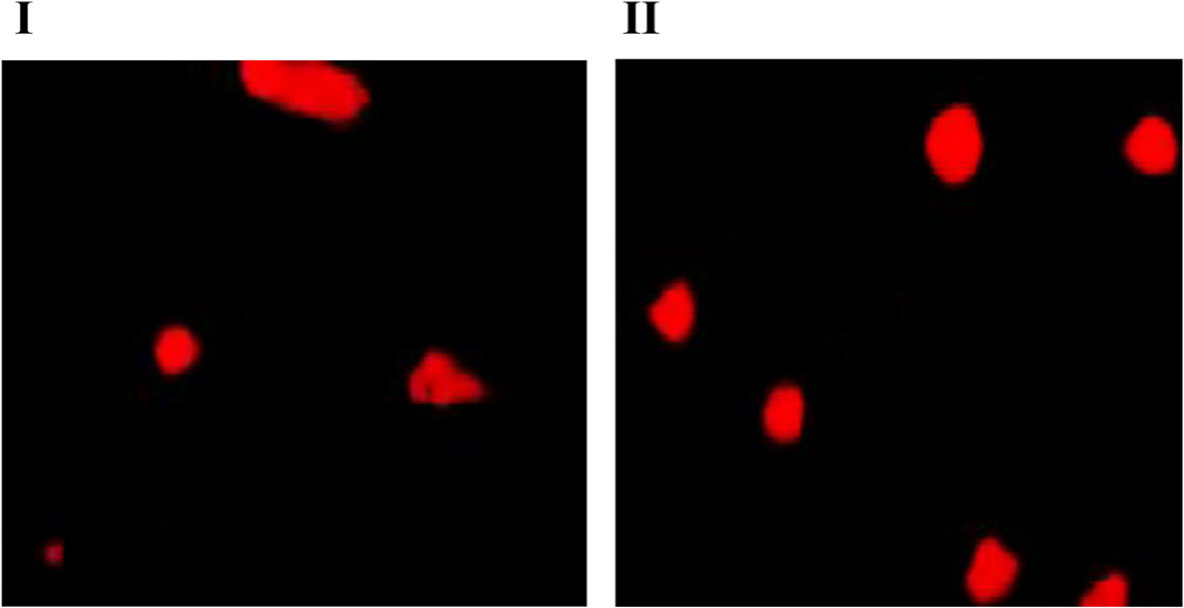
Single cell gel electrophoresis image of sperm DNA damage in the group with abnormal increase of leukocyte and the group with normal leukocyte. I. effect of abnormal increase of leukocytes on sperm DNA damage. II. sperm DNA damage in the group of normal leukocyte. The “tail” in SCGE indicates the presence of broken fragments of DNA (Magnification × 200)

T-test was used for comparison. VCL, VSL, VAP, WOB, ROS, TM, HDS, MDA, TAC showed differences between groups of abnormal/normal WBC (*P < 0.01) (Table 4, Fig. 4).

### Sperm morphology changed as seminal leukocytic level increased

**Fig. 5:**
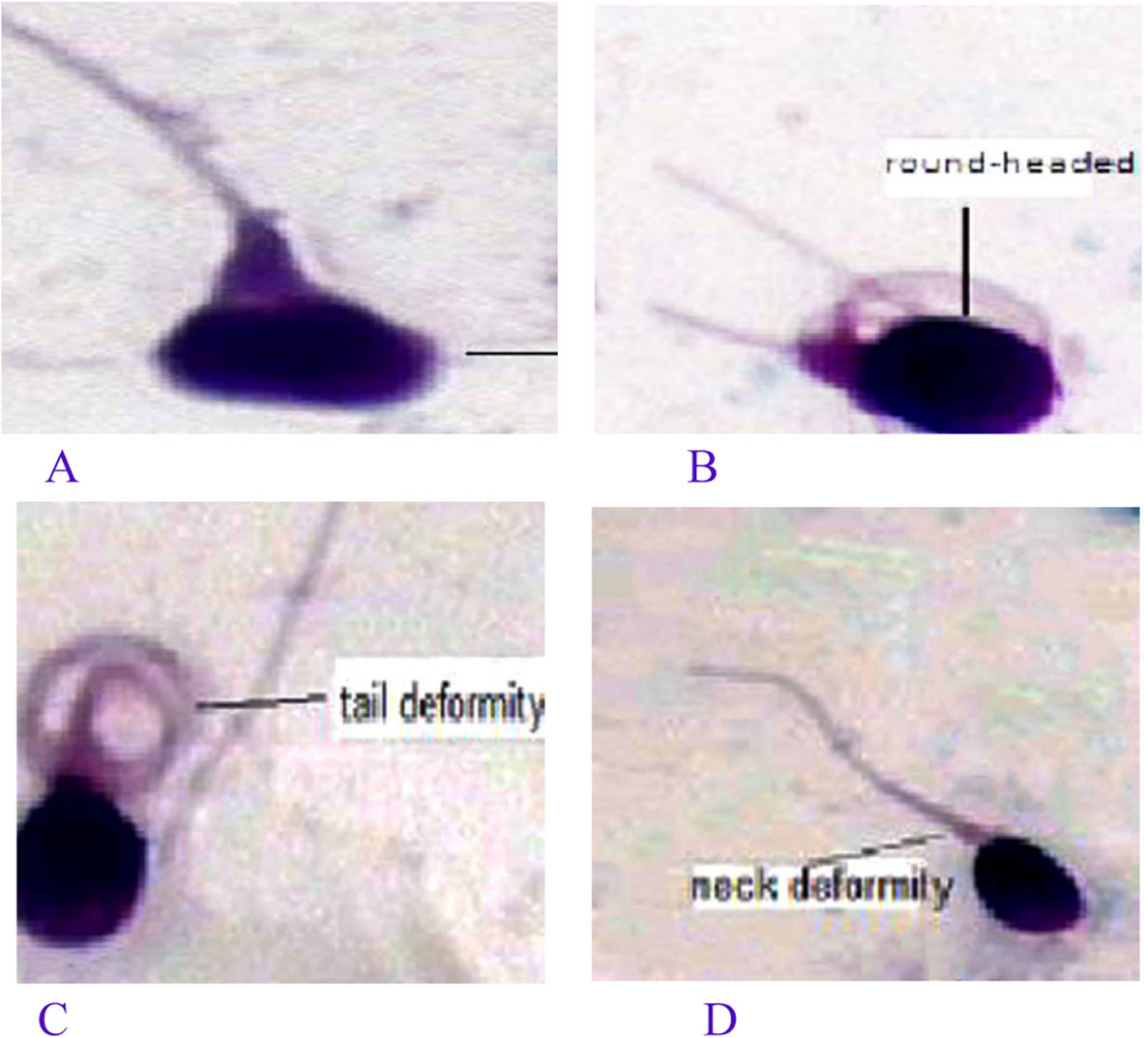
The photos of sperm deformity. **A**, **B** sperm head deformity; **C** sperm tail deformity; **D** sperm neck deformity

Seminal parameters in groups of abnormal/normal leukocytic levels were compared with t-test. Sixty normal semen samples were randomized as the control. The proportions of sperm with abnormal morphology (mostly in the head) showed difference. *P* < 0.05 was considered statistically significant (Table 5, Fig. 5).

**Table 5:** The morphology of the sperm changed as seminal leukocytic level increased

	Proportion of sperm with abnormal morphology	Proportion of sperm with Abnormal head (%)	Proportion of sperm with abnormal mid-piece	Proportion of sperms with abnormal tail %
WBC < 10^6^ (*n* = 60)	94.6 ± 5.2	75.1 ± 9.9	11.3 ± 3.8	8.3 ± 1.8
WBC > 10^6^ (*n* = 53)	96.0 ± 3.6	76.2 ± 11.8	12.1 ± 3.6	8.4 ± 2.0
t	2.1	3.1	1.1	0.6
*p*	0.005*	0.003*	0.511	0.620

## Discussion

### Effects of different reproductive tract infection groups on sperm quality and oxidative stress parameters

It is a consensus that male infertility can be caused by CT and UU infection. Most of patients who suffer from infection-induced infertility present no symptoms [13]. In Asia, the prevalence of reproductive tract infection by mycoplasma is the highest in Iran, followed by India (6.0%), and the lowest in Vietnam (0.8%). In Europe, this prevalence stays at 1.1–7.1% [30], but reaches 17.7% in China and even higher in people who have other STD or do not use condoms during sexual intercourse for more than 3 months [31]. In our present study, we compared the DFI and other routine seminal parameters in 253 patients. We found that these parameters showed significant difference between the three groups (I, II, III) and the control group, which demonstrated that CT and UU infection can affect seminal quality. In addition, CT and UU damaged DNA integrity and increased the proportion of sperm with fragmented DNA [32, 33].

Seminal elastase can be easily detected to define the infection. Elastase, a kind of soluble protease, is evenly distributed in the semen. Used as an index, elastase is more accurate, sensitive and specific than seminal leukocytes in diagnosing male reproductive tract infection, especially inapparent infection [34]. In our research study, elastase levels in Group II and Group III differed from that in Group IV, indicating that seminal vesicles and epididymides can decide the seminal volume and sperm quality. Also, the high-pH environment with an abnormal osmotic pressure increased the death rate of sperm and induced the infection of sex glands.

Male infertility can also be caused by CT-induced reproductive tract infection [35], a process of which inflammatory factors (bacteria, leukocytes) and their metabolites interact to negatively affect sperm genesis and mutation (for example, lowering the sperm density and motility) [36]. CT infection, especially in coexistence with UU infection, can worsen sperm parameters [37]. Thriving in the male reproductive tract, CT produces plenty of phospholipase A2, breaks arachilonic acid down into protaglandins, and finally triggers urethritis, epididymitis and orchitis [35]. CT can also penetrate into the sperm, destroy sperm membrane and acrosomal membrane, weaken sperm motility and inhibit the sperm from being fused into egg’s pellucid zone. In this process, the fertilization is interrupted and male infertility develops. Like UU, CT can evoke sperm autoimmune reaction. Currently, the mechanisms of CT and UU undermine the sperm quality remain unclear. In the present study, we observed that the sperm motility, progressive motility ability, vitality rate declined obviously in CT-UU-positive group when compared with CT-positive group and UU-positive group (Table 1). Previous studies have not turned to the synergistic effect of CT/UU infection on sperm quality. In fact, CT and UU act differently in damaging sperm. CT mainly damage the sperm structure, but UU mainly binds to hamper the sperm to move. Therefore, our finding may suggest their joint effects. Besides, UU only becomes pathogenic in a given condition. Therefore, once the urinary tract is damaged, sperm environment distorted and sperm structure crumbled by CT infection, UU can accelerate the decline of sperm quality.

Sperm DNA fragmentation is a useful index to assess the sperm quality. Poor sperm DNA fragmentation can reduce males’ ability to fertilize, disturb the formation of protokaryotic cells, and even induce fetal abortion, malformation or genetic diseases [38]. CT and UU infection can increase the level of DNA fragmentation. One underlying mechanism may be oxidative stress that can directly damage spermatocytes or trigger apoptosis. Besides, oxidative stress can change the organization of chromatin, the expression of proteins, and the activity of membrane transporters and receptors involved in ion channels [39]. Some analyses suggest that CT infection and UU infection do not directly decrease sperm count and motility, but increase **s**perm DNA fragmentation [40, 41]. Our research found that sperm DNA fragmentation in UU-positive group and UU-CT-positive group were statistically different from that in the control group, which is consistent with the results of previous studies [42].

In this study, the control group had an obviously lower seminal MDA level and an obviously higher seminal TAC level than the other groups, indicating that too much MDA was produced during seminal lipid peroxidation and that the drop of TAC level triggered oxidative stress reaction and destroyed the spermatic membranes [37]. According to Ni et al. [43] and Fu et al. [44], sperm DNA damage could be caused by ROS in patients with varicocele. Shang et al. [45] and Greco et al. [46] have reported that antioxidant can decrease the rate of DNA fragmentation, suggesting that the seminal ROS participates in the process of sperm DNA damage.

### Effects of different reproductive tract infection groups on leukocytes

Human semen also contains spermatogenic cells, leukocytes, and genital epithelial cells. According to the definition of WHO, once the level of seminal leukocytes exceeds 1 × 10^6^ mL/− 1, leukocytospermia arises. Compared with Group IV, the level of seminal leukocytes in Group II elevated significantly, not in the Group I. Bacteriospermia is one cause of infertility. Recent studies have demonstrated the positive correlation between bacteriospermia and leukocytospermia. However, the link between mycoplasma infection and leukocytopspermia remains blurry. It is reported that abnormal leukocytic level occurs in 43.4% of seminal samples. Besides, the bacterium-positive rate reaches 48.2% in the group of normal leukocytic level and 54.9% in the group of abnormal leukocytic level (UU being the most frequently detected bacterium). In the present study, we found that UU can increase the level of seminal leukocytes in Group II, which is consistent with the results of previous studies [47]. How seminal leukocytes affect seminal quality is still being debated. As the level of seminal leukocytes increases, the seminal volume, sperm concentration, vitality, and ability to fertilize decrease; in severer cases, the sperm chromatin may be denatured, sperm DNA fragmentation damaged, and the numbers of immature spermatogenetic cells and aberrant cells increased. In 2014, Flint et al. reported the association between leukocytospermia and oxidative stress, advocating that ROS plays a dual role: worsening the semen (abnormalizing sperm chromatin and form) and bettering the semen (energizing spermatophores and sperm to survive and move) [48]. Other studies also found that the sperm motility drops as ROS increases in patients with leukocytospermia [49]. These findings may temper the conflicts on leukocytospermia. In the present study, we found that the motility, progressive motility, and the proportion of normal sperm all decreased in the group of abnormal leukocytic level (all *P* < 0.05).

The elevated sperm ROS can lead to sperm tail damage due to cell membrane peroxidation, and change the membrane fluidity. At the same time, it can also attack the sperm nuclear DNA and cause DNA damage. Previous studies have found a 1.5 times higher level of oxidized DNA derivatives in infertile men. In order to confirm that the excessive ROS caused by abnormally increased semen leukocytes can attack and damage sperm DNA, we conducted a comparative study on the DNA damage using SCD and single-cell gel electrophoresis (SCGE). The results of SCD revealed that DFI and HDS in the patients with abnormally increased leucocytes were significantly higher than those with normal leucocyte levels. The results of SCGE showed that the TM value of the abnormal leukocyte group was significantly higher than that of the normal leukocyte group. These results indicate that the abnormal increase of semen leukocytes could cause the fracture and damage of sperm DNA and destroy its integrity and the increased ROS level might be crucial to this mechanism. Sperm DNA compacts the chromatin tightly in the nucleus and stabilizes it in a special way. This characteristic, together with the antioxidants in the spermatoplasm protects sperm DNA from oxidative attack. But because pyrimidines and purine deoxyribose are very sensitive to oxidative stress, excessive ROS can damage sperm DNA. A new research shows sperm DNA fragments are more easily detected in the semen of patients with leukocyte spermatozoosis. And leukocyte spermatozoospermia can induce sperm DNA damage, with cascade amplification effect, while excessive white blood cells are the main source of ROS production in semen [50, 51]. In this study, SCGE and SCD verified that leukocytes could damage sperm DNA by increasing ROS. Although the origin and mechanism of sperm DNA damage have not been fully understood, more and more data indicate that DNA damage is closely associated with infertility. Therefore, sperm DNA integrity could be a good indicator of fertility, as a supplement of semen parameters [52]. Excessive ROS caused by increased leukocytes in semen can lead to DNA damage, which is likely to increase the probability of male infertility (Table 4, Fig. 4).

The role of leukocytes in the semen has been well explored by the researchers around the world. Here is a to-be-proved mechanism: as the level of seminal leukocytes increases, ROS is plentifully released to ignite peroxidatic reaction in the DHA embedded in mitochondria; as a consequence, less ATP is synthesized than normal, weakening the sperm’s ability to accomplish acrosomal reaction and sperm-egg fusion [38]. In addition, the enzymes in leukocytes (peroxidase, elastase, collagenase) and their metabolites (IL-8, IFN-R, TNF) can also damage the sperm, as evidenced by the decreased motility [38]. Recent studies have confirmed that sperm DNA fragments are more easily detected in patients with leukocytospermia and may bring with cascade effects [53].

In the present study, the motile indexes of sperm (VCL, VSL, VAP, WOB) were obviously changed in the group of high-level seminal leukocytes. Studies have verified that the sperm motility decreases along with the leukocytic level increases. In the later process, the polyunsaturated fat acid (PUFA) undergoes lipid peroxidation, which in turn hardens sperm membrane and drops membrane fluidity, both decreasing the sperm motility. Besides, ROS-induced sperm membrane damage can also increase the permeability, which disables the sperm to regulate the concentration of ions involved in sperm movement; as a result, the motility deceases. Other studies have found that excessive ROS can damage the ultra-microstructure of sperm membrane and mitochondria, which also decreases the sperm motility [54].

High-level seminal leukocytes can also deform the sperm. The proportion of sperm with normal morphology significantly lowers in patients with high level of seminal leukocytes [55]. These findings verify the link between the two indicators. Overproduction of ROS can disturb the sperm-related regulatory mechanism, consequently flawing the sperm structure. In the present study, the morphological indexes showed obvious difference (most in the head) between the groups with abnormal/normal seminal leukocytic level, which is consistent with the previous findings.

Limitations also exist in this research. For instance, the sample size was comparatively small. The effect of sexual abstinence on sperm parameters was not fully considered.

## Conclusions

We found that the semen parameters showed significant difference between the three groups (I, II, III) and the control group, indicating that infection of UU and CT in the genital tract can damage seminal quality. Meanwhile, CT and UU infection damaged DNA integrity and increased the proportion of sperm with fragmented DNA. In addition, the proportion of sperm with abnormal morphology (mostly in the head) showed obvious difference between groups of high and normal seminal leukocytic levels and the motile indexes of sperm (VCL, VSL, VAP, WOB) obviously changed in the groups of high-level seminal leukocytes, revealing that the sperm motility decreases as leukocytic level increases. At the same time, SCGE and SCD verified that leukocytes could damage sperm DNA by increasing ROS. The underlying mechanisms of asthenospermia induced by UU or CT remain to be further explored.

## Data Availability

The datasets used are analyzed are during the current study are available from the corresponding author on reasonable request.

## Abbreviations

UU: Ureaplasma urealyticum
CT: Chlamydia trachomatis
DFI: DNA fragmentation index
MDA: Malondialdehyde
TAC: Total antioxidant capacity

## References

[1] Drobnis Erma Z, Angia AK (2017). Psychotropics and male reproduction. Adv Exp Med Biol.

[2] Wang JX, Shi YC (2017). Impact of mycoplasma and chlamydia infections on male reproduction. Zhonghua Nan Ke Xue.

[3] Liu J, Wang Q, Ji X, Guo S, Dai Y, Zhan Z, Jia L, Shi Y, Tai S, Lee YS (2014). Prevalence of Ureaplasma urealyticum, mycoplasma hominis, chlamydia trachomatis infections, and semen quality in infertile and fertile men in China. Urology.

[4] La Vignera S, Condorelli RA, Vicari E, Salmeri M, Morgia G, Favilla V, Cimino S, Calogero AE (2014). Microbiological investigation in male infertility: a practical overview. J Med Mi crobiol.

[5] Sergerie M, Laforest G, Bujan L, Bissonnette F, Bleau G (2005). Sperm DNA fragmentation: threshold value in male fertility. Hum Reprod.

[6] Gimenes F, Souza RP, Bento JC, Teixeira JJ, Maria-Engler SS (2014). Male infertility: a public health issue caused by sexually transmitted pathogens. Nat Rev Urol.

[7] Zhang Q, Xiao Y, Zhuang W, Cheng B, Zheng L, Cai Y, Zhou H, Wang Q (2014). Effects of Biovar I and Biovar II of Ureaplasma urealyticum on sperm parameters, lipid peroxidation, and deoxyribonucleic acid damage in male infertility. Urology.

[8] Murato S, Akarca Dizakar OS, Keskin Aktan A (2018). The protective role of melatonin and curcumin in the testis of young and aged rats. Andrologia.

[9] Sarban S, Kocyigit A, Yazar M, Isikan UE (2005). Plasma total antioxidant capacity,lipid peroxidation and erythrocyte antioxidant enzyme activities in patients with rheumatoid arthritis and osteoarthritis. Clin Biochem.

[10] Lowe PO, Loughlin P, Evans K, White M, Bartley PB, Vohra R (2006). Comparison of the GenProbe APTIMA Combo 2 assay to the AMPLICOR CT /NG assay for detection of Chlamydia trachomatis and *Neisseria gonorrhoeae* in urine samples from Australian men and women. J Clin Microbiol.

[11] Ye SZ (2002). STD Diagnosis, Treatment and Prevention [M].

[12] Li WN, Zhu WB, Liu G. Correlation of Mycoplasma genitalium infection with male infertility. Zhonghua Nan Ke Xue. 2018;24(11):999–1004. https://pubmed.ncbi.nlm.nih.gov/32212474/. 32212474

[13] Soni S, Alexander S, Verlander N, Saunders P, Richardson D, Fisher M, Ison C (2010). The prevalence of urethral and rectal mycoplasma genitalium and its associations in men who have sex with men attending a genitourinary medicine clinic. Sex Transm Infect.

[14] Black CM, Marrazzo J, Johnson RE, Hook EW, Jones RB, Green TA, Schachter J, Stamm WE, Bolan G, St Louis ME, Martin DH (2002). Head-to-head multicenter comparison of DNA probe and nucleic acid amplification tests for chlamydia trachomatis infection in women performed with an improved reference standard. J Clin Microbiol.

[15] Wang ZX, Liu H, Wu LJ (2016). Effect of infection on seminal parameters in infertile males. J Reprod Med.

[16] Papp JR, Schachter J, Gaydos CA, Barbara VD (2014). Recommendations for the laboratory-based detection of chlamydia trachomatis and Neisseria gonorrhoeae-2014. MMWR Recomm Rep.

[17] Omran HM, Bakhiet M (2013). Dashti MG.DNA integrity is a critical molecular indicator for the assessment of male infertility. Mol Med Rep.

[18] De Bantel A, Fleury-Feith J, Poirot C, Berthaut I, Garcin C, Landais P, Ravel C (2015). Simultaneous vitality and DNA-fragmentation measurement in spermatozoa of smokers and non –smokers. Cytometry.

[19] Banks S, King SA, Irvine DS, Saunders PT (2005). Impact of a mild scrotal heat stress on DNA integrity in murine spermatozoa. Reproduction.

[20] Chinese Society of Reproductive Medicine, Population and Family Planning Science and Technology Research Institute of China. Chinese Society of Andrology. In: WHO Laboratory Manual for the Examination and Processing of Human Semen. 5th ed. Beijing: People’s Health Press.

[21] Tong YY, Liu JF, Cui XL, Ma J (2016). Correlation of the inner diameter parameters of the spermatic vein in different positions and states of the varicocele patient with the results of seminal examination. Zhonghua Nan Ke Xue Za Zhi.

[22] Xu XR, Lin H, Zhang XX, Li JY, Zhang W, Sun WJ, Pan YM (2012). The effects of extremely low frequency electromagnetic field exposure on the pH of the adult male semen and the motoricity parameters of spermatozoa in vitro. Zhonghua Lao Dong Wei Sheng Zhi Ye Bing Za Zhi.

[23] Enginsu ME, Dumoulin JC, Pieters MH, Bras M, Evers JL, Geraedts JP (1991). Evaluation of human sperm morphology using strict criteria after diff-Quik staining: correlation of morphology with fertilization in vitro. Hum Reprod.

[24] Yang Y, Jiang H, Zhang H, Hong K (2012). Correlation of age,sperm DNA fragmentation with semen parameters in infertile males. Chin J Hum Sex.

[25] Wang M, Sun J, Wang L, Gao X, Lu X, Wu Z, Wang Y, Liu K, Tao J, Wu Y (2014). Assessment of density gradient centrifugation (DGC) and sperm chromatin dispersion (SCD) measurements in couples with male factor infertility undergoing ICSI. J Assist Reprod Genet.

[26] Wen-Wei C, Dun-Sheng M, Nan J, Ling-Li W (2016). Seminal elastase detection. Association of sperm DNA integrity with seminal plasma oxidative stress and its influence on in vitro fertilization in infertile males. Natl J Androl.

[27] Zhang QX, Xiao YX, Cheng BZ (2015). Impacts of leukocyte elevation on reactive oxygen species and sperm DNA damage. Carcinogen Teratogen Mutagen.

[28] Lu Y, Rong CZ, Zhao JY, Lao XJ, Xie L, Li S, Qin X (2016). Influence of storage time on DNA of Chlamydia trachomatis, Ureaplasma urealyticum, and *Neisseria gonorrhoeae* for accurate detection by quantitative real-time polymerase chain reaction. Braz J Med Biol Res.

[29] Wu YG, Yang X, Zhang H, Zheng JJ, Huang XF (2015). Impact of male reproductive tract infection on semen quality. Nat J Androl.

[30] Zhang JJ, Zhao GL, Wang F, Hong FC, Luo ZZ, Lan LN, Zhang CL, Peng Y, Liu XL, Feng TJ, Chen XS (2012). Molecular epidemiology of genital chlamydia trachomatis infection in Shenzhen, China. Sex Transm Infect.

[31] Zhang ZH, Zhang HG, Dong Y, Han RR, Dai RL, Liu RZ (2011). Ureaplasma urealyticumin male infertility in Jilin province, North-East China, and its relationship with sperm morphology. J Int Med Res.

[32] De BA, Fleury FJ, Poirot C, Berthaut I, Garcin C, Landais P, Ravel C (2015). Simultaneous vitality and DNA fragmentation measurement in spermatozoa of smokers and nonsmokers. Cytometry B Clin Cytom.

[33] Henkel R, Maass G, Jung A, Haidl G, Schill WB (2007). Age-related changes in seminal polymorphonuclear elastase in men with asymptomatic inflammation of the genital tract. Asian J Androl.

[34] Eley A, Pacey M, Galdiero M, Galdiero M, Galdiero F (2005). Can chlamydia trachomatis directlydamage your sperm. Lancet Infect Dis.

[35] Fraezek M, Kur PM (2007). In flammatory mediators exert toxic efects o foxidative stress on human spermatozoa. J Androl.

[36] Shamsi MB, Kumar R, Dada R (2008). Evaluation of nuclear DNA damage in humanspermatozoa in men opting for assisted reproduction. Indian J Med Res.

[37] Rex AS, Aagaard J, Fedder JD (2017). DNA fragmentation in spermatozoa: a historical review. Andrology.

[38] Agarwal A, Ulgund A, Alshahrani S, Assidi M, Abuzenadah AM, Sharma R, Sabanegh E (2014). Reactive oxygen species and sperm DNA damage in infertile men presenting with low level leukocytospermia. Reprod Biol Endocrinol.

[39] Gallegos G, Ramos B, Santiso R, Goyanes V, Gosálvez J, Fernández JL (2008). Sperm DNA fragmentation in infertile men with genitourinary infection by chlamydia trachomatis and mycoplasma. Fertil Steril.

[40] PanF HZ, Shi L, Chen Y, Xu ZP, Pan LJ, Dai YT (2013). Research on the relationship between sperm DNA fragmentation and seminal infection by Ureaplasma urealyticum and mycoplasma hominis of infertile males. Reprod Contracept.

[41] Chen JW, Zhang XX, Xia QQ, Cui Y (2011). Analysis of sperm DNA integrity of male infertility patients with mycoplasma infections and its application. Chin J Nosocomiol.

[42] Ni K, Steger K, Yang H, Wang H, Hu K, ZhangT CB (2016). A comprehensive investigation of sperm DNA damage and oxidative stress injury in infertile patients with subclinical, normozoospermic, and astheno/oligozoospermic clinical varicocoele. Andrology.

[43] Fu H, Song WK, Ling XH, Gao CF, Chen ZY, Zhang J, Jiang FN (2016). Correlation of oxidative stress with sperm DNA integrity and semen parameters in infertile men with varicocele. Nat J Androl.

[44] Shang XJ, Mo DS, Zhan XX, Cai HC, Ge JP, Huang YF (2015). L-carnitine protects sperm DNA damage in male infertility. J Third Mil Med.

[45] Greco E, Iacobelli M, Rienzi L, Ubaldi F, Ferrero S, Tesarik J (2005). Reduction of the incidence of sperm DNA fragmentation by oral antioxidant treatment. J Androl.

[46] de BG A, Tortolero I, Villarroel V, Molina CZ, Bellabarba CE (2000). Non sperm cells in human semen and their relationship with semen parameters. Arch Androl.

[47] Flint M, Agarwal A, du Plessis SS (2012). Leukocytospermia and Oxidative Stress/ Studies on Men’s Health and Fertility.

[48] Aggarwal R, Puri M, Dada R, Saurabh G (2017). Correlation between leukocytospermia and oxidative stress in male partners of infertile couples with leukocytospermia. Int J Reprod Contracept Obstet Gynecol.

[49] Beshay VE, Bukulmez O (2012). Sperm DNA damage:how relevant is it clinically?. Curr Opin Obstet Gynecol.

[50] Perdichizzi A, Nicoletti F, La Vignera S, Barone N, D Agata R, Vicari E, Calogero A. Effects of tumour necrosis factor-alpha on human sperm motility and apoptosis. J Clin Immunol. 2007;27(2):152–62. https://pubmed.ncbi.nlm.nih.gov/17308869/. 10.1007/s10875-007-9071-5 17308869

[51] Zini A, Albert O, Robaire B (2014). Assessing sperm chromatin and DNA damage: clinical importance and development of standards. Andrology.

[52] Robinson L, Gallos ID, Conner SJ, Rajkhowa M, Miller D, Lewis S, Kirkman-Brown J, Coomarasamy A (2012). The effect of sperm DNA fragmentation on miscarriage rates:a systematic review and meta-analysis. Hum Reprod.

[53] Menkveld R, Holleboom CA, Rhemrev JP (2011). Measurement and significance of sperm morphology. J Androl.

[54] Aziz N, Lewis-Jones DI, Agarwal A, Saleh RA, Sharma RK, Thomas AJ (2003). Sperm morphology and seminal leukocytes as predictors of increased production of reactive oxygen species (ROS) in infertile men semen. Fertil Steril.

[55] Wolff H, Bezold G, Zebhauser M, Meurer M (1991). Impact of clinically silent inflammation on male genital tract organs as reflected by biochemical markers in semen. J Androl.

